# Role of Autophagy in Lung Inflammation

**DOI:** 10.3389/fimmu.2020.01337

**Published:** 2020-07-07

**Authors:** Jacob D. Painter, Lauriane Galle-Treger, Omid Akbari

**Affiliations:** Department of Molecular Microbiology and Immunology, Keck School of Medicine, University of Southern California, Los Angeles, CA, United States

**Keywords:** autophagy, asthma, lung inflammation, immunometabolism, COVID-19, SARS-CoV-2

## Abstract

Autophagy is a cellular recycling system found in almost all types of eukaryotic organisms. The system is made up of a variety of proteins which function to deliver intracellular cargo to lysosomes for formation of autophagosomes in which the contents are degraded. The maintenance of cellular homeostasis is key in the survival and function of a variety of human cell populations. The interconnection between metabolism and autophagy is extensive, therefore it has a role in a variety of different cell functions. The disruption or dysfunction of autophagy in these cell types have been implicated in the development of a variety of inflammatory diseases including asthma. The role of autophagy in non-immune and immune cells both lead to the pathogenesis of lung inflammation. Autophagy in pulmonary non-immune cells leads to tissue remodeling which can develop into chronic asthma cases with long term effects. The role autophagy in the lymphoid and myeloid lineages in the pathology of asthma differ in their functions. Impaired autophagy in lymphoid populations have been shown, in general, to decrease inflammation in both asthma and inflammatory disease models. Many lymphoid cells rely on autophagy for effector function and maintained inflammation. In stark contrast, autophagy deficient antigen presenting cells have been shown to have an activated inflammasome. This is largely characterized by a T_H_17 response that is accompanied with a much worse prognosis including granulocyte mediated inflammation and steroid resistance. The cell specificity associated with changes in autophagic flux complicates its targeting for amelioration of asthmatic symptoms. Differing asthmatic phenotypes between T_H_2 and T_H_17 mediated disease may require different autophagic modulations. Therefore, treatments call for a more cell specific and personalized approach when looking at chronic asthma cases. Viral-induced lung inflammation, such as that caused by SARS-CoV-2, also may involve autophagic modulation leading to inflammation mediated by lung resident cells. In this review, we will be discussing the role of autophagy in non-immune cells, myeloid cells, and lymphoid cells for their implications into lung inflammation and asthma. Finally, we will discuss autophagy's role viral pathogenesis, immunometabolism, and asthma with insights into autophagic modulators for amelioration of lung inflammation.

## Introduction

Eukaryotic organisms need to break down intracellular constituents for a variety of reasons. These constituents can range from microbial invaders to their own cellular components, such as organelles and proteins. In the case of microbial invaders, these cells want to seek and specifically kill these smaller cells to protect themselves from infection (1, 2). However, destruction of their own cellular components is a highly regulated cellular process triggered by a variety of stimuli (1, 3, 4). This process of cellular recycling and degradation was coined autophagy in 1963 (5, 6). The name which directly translates from Greek meaning “self-eating.” Common induction pathways of autophagy include stress and starvation signals. Dysregulation and malfunction of this crucial cellular component has more recently gained attention in the pathogenesis of many types of disease (1).

There are several types of autophagy in which cytosolic components are sent to the lysosome for degradation. Macroautophagy is the most prevalent form of autophagy in the cell and is responsible for much of the organelle and microbial degradation; this is through formation of a double membrane vesicle which surrounds the component and fuses to the lysosome (7, 8). This vesicle is called an autophagosome and when fused with the lysosome it becomes an autolysosome containing all the hydrolytic enzymes needed to break down its contents (4). Besides cellular components, autophagosomes can include macromolecules, such as lipids, sugars, nucleic acids, and proteins. The role of macroautophagy has been also shown to play a central role in immune function as knockout of autophagy genes in *Drosophila* make them more susceptible to both viral (vesicular stomatitis virus) and bacterial (*L. monocytogenes*) infection (9, 10). The second type of autophagy, microautophagy, deals with the degradation of cellular components largely without formation of an autophagosome. It is largely defined by the engulfment of cytoplasm and budding into the lumen of the lysosome (11, 12). The third type of autophagy is called chaperone-mediated autophagy (CMA). This type of autophagy relies on chaperones to mediate the transport of components to the lysosome for degradation; a large part of this system is reliant on lysosome-associated membrane proteins (LAMPS) for proper component processing (13, 14). These three distinct systems make up one of the most important survival systems in eukaryotic organisms and even without stimulus are still present at basal levels. Due to most studies focusing on macroautophagy, it will now be referred to as autophagy unless otherwise specified.

Regulation of autophagy is a complex cellular process involving a variety of different proteins. In this review, the models used utilize a variety of methods of autophagy-deficiency through depletion or deletion of these important proteins or genes. The general mechanism of autophagy and autophagosome formation involves three steps: initiation, nucleation, and elongation (15). The involvement of sixteen or more autophagy-related genes (Atg) products have been characterized in these three processes (15). Although there are many proteins involved in autophagy, the majority of studies covered in this review, involving genetic knockouts or otherwise, utilize autophagy-related gene 3 (Atg3), autophagy-related gene 5 (Atg5), and autophagy-related gene 7 (Atg7). These protein products are involved in the elongation step of autophagosome formation and differ in their roles (15). In the elongation step, Atg5 and autophagy-related gene 12 (Atg12) are conjugated and associated with autophagy-related gene 16 like 1 (Atg16L1) and microtubule-associated protein 1A/1B-light chain 3 (LC3) to promote autophagosome formation (15, 16). Atg7 is involved in catalyzing the process of Atg12-Atg5 conjugation (17). Atg3 and Atg7 are involved in the lipidation process of LC3 with phospholipid phosphatidylethanolamine (PE) and are necessary for LC3 function and therefore autophagosome elongation (17). Atg3 can also indirectly affect formation of Atg12-Atg5 conjugation as mature LC3 seems to be needed for Atg12-Atg5-Atg16 complex formation (17), and Atg3-deficient mice show dramatically reduced Atg12-Atg5 conjugation (18). Therefore, in studies involving genetic modulation of Atg3, Atg5, and Atg7, it can be noted that loss of function in these proteins halt the elongation step of autophagosome formation and can be known as autophagy-deficient at some level. However, there is controversy onto what level autophagy is affected depending on what gene is being studied. In the case of Atg3, Atg5, and Atg7 mouse models, the functionality of the protein products must be maintained through birth as deletion of these genes results in neonatal lethality which points to their essential role in autophagy function *in vivo* (19). The deletion of many Atg protein products result in autophagy-deficiency which can be studied for various applications including lung inflammation (19).

Autophagy plays a key role in cellular function of a variety of different immune cell types. For example, in myeloid cells, due to the processing of antigen it is logical that autophagy would be involved heavily in these pathways. Autophagy has been since been described to play pivotal roles in a variety of different myeloid cell types. Many neutrophil functions have been linked to autophagy including differentiation (20), extracellular trap formation (21, 22), and cytokine secretion and interaction (23, 24). Eosinophils, important in allergic disease, have also been demonstrated to have diminished eosinophil extracellular trap formation (EET) when autophagy is inhibited (25). Extensive use of autophagy has also been observed in antigen presenting cells (APCs). Dendritic cells use autophagy extensively for a variety of functions including but not limited to: MHC class II antigen presentation, cytokine secretion, and activation of lymphoid cells (26). In particular, autophagy deficient macrophages have been shown to exacerbate eosinophilic inflammation through PGD_2_ dysregulation (25). It is possible that this dysregulation may also lead to the recruitment of lymphoid cells, such as T cells and type two innate lymphoid cells (ILC2). Recently our lab has demonstrated that autophagy plays a critical role in the effector function of ILC2s (27). Autophagy has been shown to play a key role in a variety of T cell functions including differentiation, metabolism, survival, and activation (28). The role of autophagy within the network of these immune cells and their functions display potential for therapeutic approaches to target them for amelioration of inflammatory disease symptoms.

Due to its wide scope, autophagy has become a subject of interest in the pathology of many diseases and disorders. Disruption of these normal pathways is currently being investigated as a causative factor variety of inflammatory and allergic diseases, such as asthma and airway hyperresponsiveness (AHR). The most common treatment option for asthma is the use of short-term inhaled corticosteroids and long-term β_2_-agonists for relaxing airway smooth muscle (ASM), however these are not recommended for all chronic cases and have been found to be ineffective on 10–15% of patients (29–31). This is a significant number of the asthmatic population that is unaffected by these common treatments. Although it is a small percentage overall, these patients make up ~50% or more of asthmatic related health costs (32). Treatments seeking to target the immune cells rather than ASM itself may provide a long-term and more effective therapeutic target. Allergic asthma is largely mediated by T_H_2 cells and type two innate lymphoid cells (ILC2) for production of inflammatory cytokines IL-5 and IL-13 (33–35). Chronic cases are largely due to secondary factors associated with T_H_17 cell mediated neutrophilic inflammation (34, 36). Autophagy has been shown to play key roles in neutrophil mediated inflammation (21, 37). It is estimated that later stage chronic asthma has little to no T_H_2 cell mediated inflammation (38). This is hypothesized to be the cause of ineffective β_2_-agonist treatment on chronic asthma cases which should be effective on T_H_2 asthma phenotypes. There is also a major role that airway remodeling plays in the ineffectiveness of these treatments as well (39, 40). Understanding the multiple aspects of the pathogenesis of pulmonary inflammation in these patients is key to finding clinical treatments.

Elevated levels of autophagosome formation have been reported in peripheral blood cells as well as airways in chronic asthma patients (41, 42). Recently autophagy has been shown to play a key role in T_H_17 mediated asthma in APCs (43). This mechanism hints to possible causes of chronic asthma through the dysregulation and disruption of these important homeostatic pathways. Autophagy has also been observed to play a vital role in eosinophil mediated asthma as well (41, 44). Interestingly, correlations have also been drawn between high genetic polymorphisms of Atg5 in both adult and childhood asthma patients (45, 46). Emphasizing autophagy's role in a variety of inflammatory diseases, associations between Atg16L1 polymorphisms and irritable bowel disease have also been observed (47). Dysfunctional autophagy pathways also lead to metabolic remodeling that leads to airway inflammation (28, 30). Epithelial cells are also involved in exacerbating pulmonary inflammation and are available therapeutic targets (48, 49). Autophagy plays a key role in the regulation of cytokine signaling in a variety of cell types, likely leading to the pathogenesis of pulmonary diseases.

Autophagy involvement in the pathogenesis of many diseases makes it an important field of interest for clinical treatments for chronic patients. Making connections between its dysfunction in different cell types is crucial to understanding its role in these diseases. Because the overexpression or under expression of autophagic pathways has different effects on each cell type, understanding the role of autophagy in each individual cell type is needed to understand the network as a whole. The role of autophagy is different in each cell type which makes systemic treatments through autophagy for lung inflammation challenging. Contributions of autophagy in cellular metabolism also need to be discussed as it is important in the understanding of lung homeostasis. External factors, such as viruses are also relevant due to their regulation of autophagy contributing to lung remodeling and inflammation. Characterization of autophagy and its effects on myeloid, lymphoid, and epithelial cells in pulmonary systems will be explored in this review for its insights into clinical treatments of lung inflammation and asthma.

## Role of Autophagy in Non-Immune Cells

Previously thought to be innocent bystanders in disease pathogenesis, cells lining the pulmonary airways, such as epithelial and mesenchymal cells have also been described to be involved in inflammatory response. Autophagy has been identified in many different cases to be a major player in the effector function of these cells. In response to cytokines, such as IL-13, it has been demonstrated that autophagy is induced in epithelial cells resulting in mucus secretion (50). Interestingly, IL-13 stimulation of pulmonary epithelial cells also showed the dependence of superoxides on autophagy levels; correlations were also found with IL-4 (51). Formation of superoxides in pulmonary airways can further lead to oxidative stress in tissues and autophagy itself. In the context of lymphoid cells, typically T_H_2 cytokines inhibited autophagy while T_H_1 cytokines were activating, further suggesting that autophagy needs to be studied on a cell-to-cell basis. Further adding to cell-context dependence, it has been established that IL-17A, a T_H_17 cytokine, inhibits autophagy in lung epithelial cells through BCL2 degradation (52). In the stimulation of lung epithelial cells with interferon-γ (IFN-γ), autophagy was induced and was shown to control annexin A2 exomal release (exophagy) (53). These findings also suggest that there is not only cell-context dependence but also a possible cytokine dependence as well. Other sources have observed increase in autophagy in response to IFN-γ with different epithelial cell populations (54, 55). These observations could be related to the antiviral function of IFN-γ where it would induce cells to clear viral load. It has also been reported that annexin A2 could stimulate plasminogen mediated inflammatory cytokine production, mainly IL-6, by airway smooth muscle cells (56). This is an interesting situation where autophagy would impact T_H_2 mediated inflammation through indirect pathways. Often it is these indirect pathways in which autophagy can have a significant effect on immune response. On the contrary, it is these same indirect pathways that make targeting of autophagy for clinical applications challenging.

Autophagy contribution in the pathogenesis of a variety of inflammatory diseases through epithelial cells is implicated in the literature. It has been described by multiple sources that attenuated or impaired autophagy leads to epithelial cell dysfunction and lung fibrosis (57). This is likely due to loss in their role of secreting both anti-fibrotic and profibrotic mediators. TGF-β, a key fibrotic modulator secreted by epithelial cells, has also been demonstrated to control autophagic activity in myofibroblasts depending on the tissue and inflammatory context (58, 59). This is due to the gene transcription in response to TGF- β being cell type, cellular condition, and microenvironment dependent. Epithelial cells in pulmonary airways are key in regulation of lung homeostasis, and maintaining healthy populations of these cells are critical for avoiding lung inflammation. Autophagy has been determined to be essential in the maintenance of epithelial cell counts in pulmonary airways (60). Loss of epithelial cells can happen through a variety of different pathways, but the most common is particulate inhalation. In protection against these particles, human bronchial epithelial cells have been found to increase mucus secretion through autophagy pathways (61). Cell death due particulates can also release inflammatory factors related to pulmonary disease. Autophagy therefore can have a direct protective role on epithelial cell populations.

Interestingly, autophagy also has been shown to play a role in airway remodeling as well. Remodeling of pulmonary airways has long lasting and irreversible effects on asthmatic patients. In remodeled asthmatic patients, epithelial thickening, reticular basement membrane, and increased airway smooth muscle bundles all important airway remodeling markers, have been observed; these patients have been described to have decreased tissue inflammation with intranasal administration of chloroquine, a known autophagy inhibitor (62). In house dust mite (HDM) induced murine allergic asthma models, autophagy inhibition with chloroquine also reduced concentration of TGF-β1 in bronchoalveolar lavage (BAL) and prevented bronchoconstriction (62). This inhibition provides a type of early treatment option for patients who may have not experienced full lung remodeling. However, regulation of autophagy by TGF-β comes on a cell to cell basis (63). Smooth muscle is also involved in the remodeling of pulmonary airways (64). Extracellular matrix depositions, such as collagen formation between muscle cells can increase bundle mass and fibrosis (65). TGF-β, and its downstream mediators, are major contributors to the maintenance and promotion of fibrosis and collagen production in these tissues (66). Interestingly there are two general hypotheses and support for both autophagy increasing and decreasing fibrosis and inflammation (67). However, the basis of these hypotheses could be based in two different contexts; these include genetic background, environment, and stage of conditioning. For example, the conditioning of one patient may result in autophagy mediated tissue fibrosis in their pulmonary airways while another patient conditioned another way may result in autophagy inhibiting fibrosis. The stage of disease is critical in understanding the role autophagy plays in pulmonary tissues and further explorations into these interactions are required.

## Role of Autophagy in Myeloid Cells

### Granulocytes

The role of autophagy in myeloid cells is crucial for multiple functions in those cells. The overexpression or underexpression of this mechanism can have various effects depending on cell types (Table 1). In the case of granulocytes, such as neutrophils, they extensively use autophagy for a variety of essential functions, several which are implicated for inflammatory responses. Autophagy has a predominant role in degranulation which is one of the main functions of neutrophils (37). It is the process where neutrophils secrete cytoplasmic granules containing preformed antimicrobial and inflammatory proteins. Dysregulation of this process can lead to chronic disease due to constant tissue damage due to inflammatory proteins. Interestingly, knockout of Atg5 in neutrophils provided no evidence of abnormalities, such as granule proteins, apoptosis markers, migration, or effector functions (20). However, when myeloid-specific Atg5 and Atg7 autophagy-deficient murine models were tested, the results showed an actual decrease in neutrophil-mediated inflammatory and autoimmune disease models (37). These observations demonstrated that specific neutrophil knockout of Atg5 is not able to consistently reduce inflammation. Extracellular trap formation by neutrophils is also a major function in response to microbial invaders but also plays a key role in inflammatory response. Abnormal extracellular trap formation has been reported to have implications in a variety of autoimmune and autoinflammatory diseases reviewed by Delgado-Rizo et al. (80). It has been shown that lungs of asthmatic patients have increased migration of neutrophils and eosinophils to the lungs, which then exhibit extracellular trap formation (81). These neutrophil extracellular traps could cause a large amount of damage to airway epithelium as well as trigger responses by both airway epithelial cells and peripheral blood eosinophils (21). These interactions are what exacerbate the cycle of airway hyperreactivity and bronchial constriction associated with the pathology of asthma. Even though it has been established that Atg5 is not necessary for extracellular trap formation (82), multiple studies have noted abnormal extracellular trap formation in autophagy-deficient models (21, 22). Autophagy also does play a role in the priming of neutrophils for NET formation (83) and Atg7 knockouts have been determined to significantly affect NET formation (37). In the literature there is a current debate on how much involvement there is between autophagy in NET formation as there is evidence for both hypotheses (84). Neutrophils have been observed to conduct a caspase-independent form of cell death through an autophagy dependent pathway; this observation suggests a possible protective role in inflammatory contexts where it would encourage apoptosis of activated neutrophils. This mechanism is largely cytokine dependent, neutrophils exposed to GM-CSF and inflammatory cytokines went through an autophagy-dependent caspase-independent cell death associated with autophagosome formation (85). Moreover, Atg5 knockdown in neutrophils showed decreases in proinflammatory cytokines, such as IL-1β (23). Targeting neutrophil autophagy in certain phenotypes of asthma may provide a clinical avenue for treatment, however knockdown may decrease inflammation mediated through NET formation while also promoting neutrophil survival. Therefore, modulation of autophagy function in asthmatic patients does provide a treatment option in neutrophil mediated inflammation in severe asthma and other inflammatory diseases.

**Table 1:** Summary table of autophagy in myelocytes.

**Autophagy-related gene**	**Cell type**	**Function of autophagy**	**References**
Atg5	Neutrophils	IL-1β secretion	(23)
	Dendritic cells	Inflammatory homeostasis	(30, 68, 69)
		MHC class II antigen presentation	(70–73)
		MHC class I internalization	(72, 74)
		pDC cytokine production	(75)
	Macrophage	Inflammatory homeostasis	(68, 69, 76–78)
		Monocyte differentiation	(71)
Atg7	Neutrophils	Extracellular trap formation	(37)
	Dendritic cells	Inflammatory homeostasis	(25)
	Macrophages	Inflammatory homeostasis	(25, 79)

Autophagy has a significant role in eosinophil activation and effector function, however there are still many questions to be investigated. Eosinophils are critically involved in most asthma cases as they are recruited by the T_H_2 mediated response. It has been demonstrated that eosinophils are activated by the presence of IL-5. Eosinophil activation induces autophagy and the production of eosinophil cationic protein (ECP) further leading to inflammation (41). Autophagy also likely contributes in increasing survivability of eosinophils. ECP secretion has a variety of different effects on surrounding cells, such as inflammation and mucus hypersecretion which leads to airway constriction. Interestingly it was found that knockout of autophagy, Atg7^−/−^, in myeloid cell lineages showed an overall increase in eosinophils, epithelial hyperplasia, and mucosal thickening in eosinophilic chronic rhinosinusitis (25). This was largely characterized by the large increase in PGD_2_ which is responsible for the inflammatory recruitment of many types of immune cells myeloid and lymphoid alike. This suggests that specific targeting of cell types is needed rather than just lineage specific or systemic knockdown. The seeming contrast between these two findings is possibly explained through the differences in disease, however IL-5 exposure may play a more predominant role than just increased autophagy in severe asthma patients. Eosinophil extracellular trap formation has also been found to be correlated with autophagy levels (21, 22), but interestingly Atg5 knockout was not necessary for their formation (82). Autophagy may have a role in the differentiation of eosinophils, however the only study of it has been through regulators, such as mTOR which regulates it indirectly (86, 87). Like neutrophils, eosinophils have also been observed to go through an autophagy-mediated caspase-independent cell death in inflammatory conditions (88). Eosinophils also are major producers of TGF- β which is heavily involved in airway remodeling and early stages of chronic asthma development (63, 89). Most findings demonstrate that there are correlations in levels of eosinophil-mediated inflammation and autophagy, however, further explorations are required to draw connections for clinical applications. Due to being one of the main cell types associated with asthma and allergic diseases, characterizing the role of autophagy in eosinophils will open avenues for clinical targets. With more study and understanding, targeting of autophagy in eosinophils in T_H_2 phenotypes of asthma may ameliorate lung inflammation.

### Antigen Presenting Cells

Autophagy is extensively utilized in dendritic cells (DCs) and solving its mechanism of action has possible implications for understanding lung inflammation and asthma. Autophagy and its interactions with cytokine production in dendritic cells has largely been reviewed by Harris in bacterial and viral infection (90), however its role in inflammatory disease has largely remained unexplored. In DCs derived from peripheral blood mononuclear cells (PBMCs), the inhibition of autophagy showed reduced levels of IL-10 production leading to the proliferation of T-cells (91). Regulatory T cells have also been demonstrated to downregulate autophagy in DCs which helps mediate inflammatory responses (92). Implication of these findings could be related to allergic disease; however, it has not been fully characterized. The mechanism of autophagy has also been described to regulate plasmacytoid dendritic cells (pDCs) activation. Cytokine production, mainly interferon-α (IFN-α), secreted by plasmacytoid dendritic cells is affected by Atg5 knockout (75). Functional processing of TLR7 viral antigens also requires autophagy and without Atg5, cytosolic viral replication intermediates fail to be transported into the lysosome. Therefore, autophagy has a key role in mediating antiviral response through viral ssRNA detection and subsequent cytokine response (75). Cell-specific Atg5-deletion in CD11c^+^ cells, was found to augment lung inflammation with increased IL-17A levels in pulmonary airways leading to severe neutrophilic asthma in mice with and without HDM-challenged (30). The role of autophagy in antigen processing in DCs is the most prevalent in the literature, and improper allergen presentation leads to inflammation in multiple tissue types. Presentation to CD8^+^ T and CD4^+^ T cells through class-I and class-II MHC formation, respectively involves autophagy. The process in which autophagy proteins are involved in antigen presentation has been previously reviewed in the physiological context (93) so its relation to allergic disease will be described here. Atg5 has been shown to be required for MHC II antigen presentation as it is required for optimal phagosome-to-lysosome fusion. However, the same knockout did not affect MHC I presentation negatively (70). In inflammatory murine models of encephalomyelitis, Atg5 expression is required for MHC II myelin antigen presentation leading to disease pathogenesis (71). This suggests possible implications into other inflammatory diseases, such as asthma. However, knockout of autophagy, Atg5 and Atg7, has also been shown to increase MHC I antigen presentation enhancing the CD8^+^ T cell responses to infection *in vitro* and *in vivo* (74). This induction was due to disrupted internalization of MHC I molecules which is regulated by normal autophagy pathways. Increased MHC class I presentation would be able to further polarize T cell effector response in allergic contexts, such as increased T_H_17 polarization found in Suzuki et al. (30). It seems that autophagy machinery has more direct regulatory control over MHC II-restricted antigen presentation and indirectly controls MHC class I expression. Parallels can also be drawn where this antigen presentation could also increase the severity of inflammatory diseases through this increased T cell response. CMA has also been implicated to promote antigen presentation on APCs' surfaces and generate hyperactive CD4^+^ T cells in systemic lupus erythematosus (SLE) models (94). CMA involvement in immunity has largely been unexplored as well as autophagy's role in antigen presentation in the context of asthma.

In CD11c-specific Atg5^−/−^ murine models, CD11c^+^ DCs were determined to be the cause of unprovoked neutrophilic asthma without need for HDM challenge (30). This demonstrates the importance of autophagy in the role of APCs in inflammatory contexts. These results also were found to be DC specific as Atg5^−/−^ in pulmonary epithelial cells showed similarities to wild type (WT). Sublethally irradiated WT mice were inoculated with bone marrow (BM) cells isolated from Atg5^−/−^ mice and developed significantly high AHR (30). These results indicated that BM-derived Atg5-deficient immune cells were the cause of severe AHR rather than other non-hematopoietic cells. Chimeric experiments inoculating irradiated Atg5^−/−^ mice with WT or Atg5^−/−^ bone marrow confirmed the same results with the role of autophagy in DCs (30). Further analysis with confocal microscopy revealed asthma reduced number of LC3 foci in pulmonary dendritic cells. Bone marrow derived dendritic cells were described to increase concentrations of IL-1 and IL-23 in Atg5^−/−^ mice further contributing to T_H_17 neutrophilic polarity (30). In response to steroid treatment, Atg5^−/−^ mice were found to have no decrease in AHR showing a steroid resistant phenotype (30). As previously stated, neutrophilic asthma may account for steroid resistance in some asthma phenotypes. Challenge with HDM also demonstrated the same neutrophilic asthma phenotype as the WT suggesting that the initiation of asthma in Atg5^−/−^ mice does not need to be provoked with allergen (30). This study establishes the significant impact that genetics can have in the unprovoked asthma response in patients. The role of autophagy in the pathogenesis of neutrophilic asthma is prevalent and needs more study to fully understand different phenotypes (30). In conformation of these findings, metabolic shift of APCs has been described to promote neutrophilic inflammation in the lungs through mTOR ablation, a key regulator of autophagy (43). The role of autophagy in the reprogramming of APCs into a more inflammatory phenotype can be a pivotal point of clinical interest.

**Figure 1:**
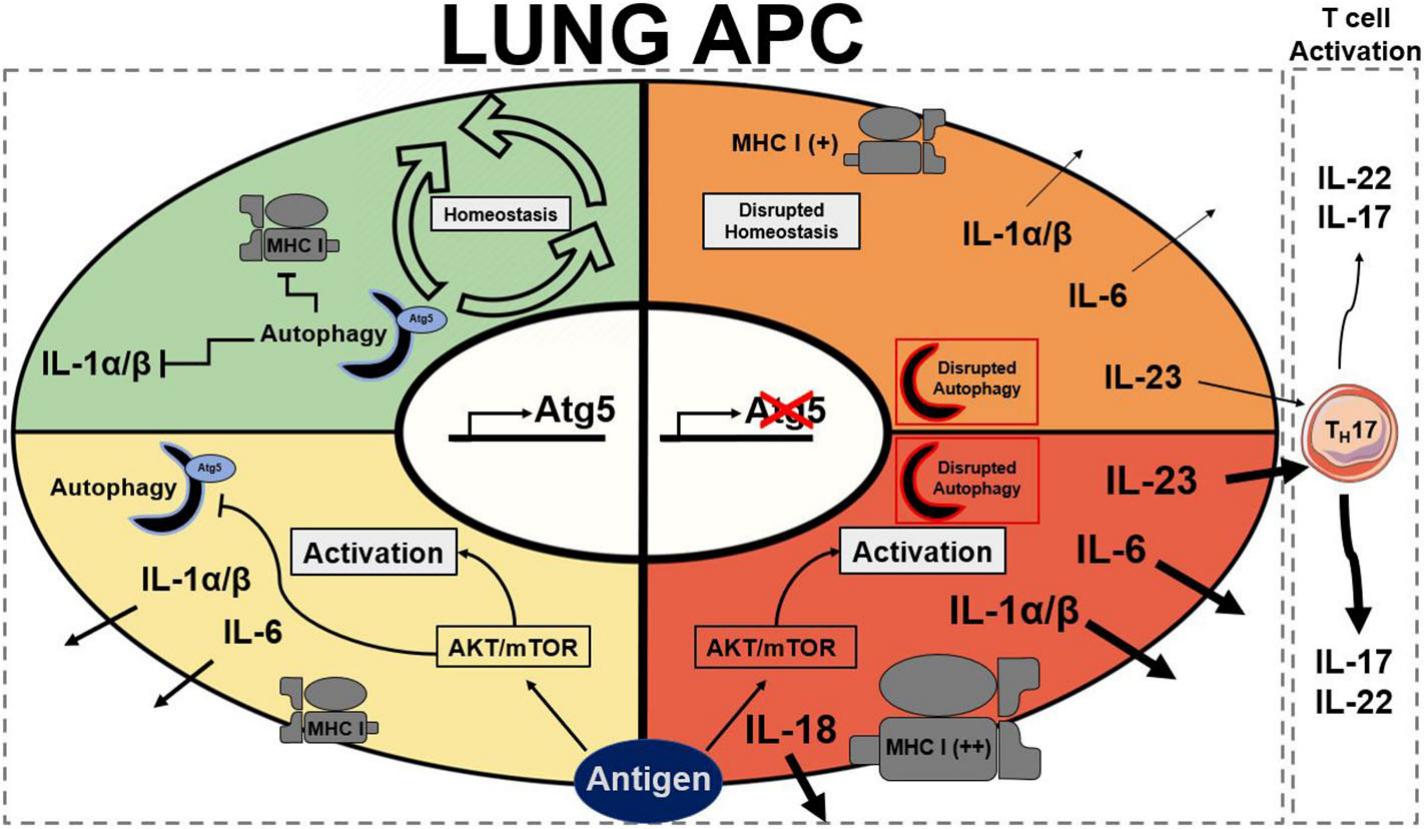
Summary of stead state and active Atg5/Atg5^−/−^ antigen presenting cells. Autophagy presence is represented by a half formed autophagosome highlighted in blue for functional and red to dysfunctional autophagy. Atg5 is a requirement for functional autophagy. Each quadrant denotes activation state of a lung APC: green represents homeostasis, yellow represents moderate activation, orange represents moderate to high activation, and red represents high activation. AKT, protein kinase B; APC, antigen presenting cell; Atg5, autophagy related 5; MHC I, major histocompatibility complex class I; mTOR, mechanistic target of rapamycin (25, 30, 74, 77, 79, 96).

Like DCs, macrophages have also been implicated with autophagy in the literature as they are also APCs. Metabolic disturbances, mTOR ablation, in macrophages have been described to directly initiate neutrophilic asthma (43). As previously mentioned, the connections between autophagy and mTOR are extensive. Autophagy also plays important roles in the differentiation of monocytes into macrophages through colony stimulating factor-1 (CSF-1) (95); further support showed that GM-CSF administration blocked cleavage of Atg5 and promoted autophagy, and without it differentiation of macrophages was attenuated along with cytokine secretions (96). Previous studies reported that hampered autophagy, through blocking of Atg5/7, LC3, and Beclin-1, led to increases in IL-1β and IL-18, suggesting a possible protective role of autophagy in inflammatory contexts (25, 79, 96). Autophagy-deficient mice with myeloid-specific deletion of Atg5 and Atg7 with sterile lung inflammation were also shown to have increased goblet metaplasia and collagen concentrations driven by IL-18 secretion (97). In macrophages, the clearance of damaged mitochondria through autophagy is very important for these reasons. Interestingly, knockout of autophagy increased antiviral resistance but also increased inflammation and cytokine secretion (76). In obese mice models, the attenuation of autophagy in macrophages was associated with increased inflammation and acute liver injury (98, 99). Atg5 has also been reported to explicitly suppress production of IL-1β (77). In models for Crohn's disease, a prevalent autoimmune disease, a mechanistic link between macrophage autophagy and the systematic disease has been identified (100). It has also been shown that dysfunctional lysosomal and autophagic mechanisms leading to inflammasome activation in macrophages (101). In inflammatory contexts, it is important to maintain autophagy to protect against inflammasome mediated IL-1β secretion and subsequent inflammation mediated by macrophages. Effect of Atg5 knockout in APCs is summarized in Figure 1. Possible connections between these inflammatory disease phenotypes may be applicable for investigation into asthma phenotypes. The production of IL-1 has been proven to exacerbate neutrophilic asthma (30). Although T_H_1 cytokines secreted by macrophages have been characterized, the subsequent production of T_H_2 cytokines by activated T cells has been unexplored with its connection to APC autophagy. In allergic contexts, the production of colony stimulating factors increases secretion of cytokines by macrophages, however the following stimulation of T_H_17 cells has yet to be explored. GM-CSF has found shown to be necessary for the maintenance of allergic asthma (102) showing that macrophage targeting with GM-CSF to induce autophagy is not a viable clinical treatment. Past studies have established that levels of GM-CSF are highly elevated in the BAL of asthmatic patients compared to healthy controls (103). These observations clearly demonstrate that other effects of GM-CSF on other cell types overpowers the macrophage inflammasome suppression effect. Therefore, preservation of the autophagic flux in macrophages must be done through other means to avoid inflammasome activation. Antigen presentation by macrophages after increased monocyte differentiation by GM-CSF could impact pulmonary inflammation through subsequent activation of T cells. Overall preservation of autophagy in macrophages is essential for suppression of the inflammasome. However, there are still open questions requiring further exploration regarding the role of autophagy in macrophages, specifically involving antigen presentation and cytokine production.

## Role of Autophagy in Lymphoid Cells

### Lymphocytes

The role of autophagy in CD8^+^ T cells show the wide scope of its contribution in multiple cell types (Table 2). It has been demonstrated that autophagy plays a key role in various functions of CD8^+^ T cells, but more specifically the proliferation, metabolism, survival, and memory function (109, 110). CMA has been implicated into the function of T cells as it specifically degrades negative regulators of T cell activation (127). Multiple early studies have observed lower CD8^+^ T cell counts in spleen and lymph nodes with increased apoptosis in Atg3, Atg5, and Atg7 deficient murine models (104, 107, 128). As these cells replicate in an inflammatory context it makes sense that autophagy is upregulated to remove unwanted reactive oxygen species (ROS) and to clear defective mitochondria through mitophagy. In these highly replicative states, involvement of mTOR is extensive as the need for protein synthesis is high (129). The interplay between mTOR and autophagy in CD8^+^ T cells is crucial to understanding their function. The canonical model of their interaction is that mTOR is a negative regulator of autophagy (130); this is largely based on their differing roles in protein synthesis and protein recycling, respectively. However, some studies have found that this is not always the case in all CD8^+^ T cell subsets (131). Further adding to the complexity of CD8^+^ T cells is the metabolic reprogramming that they undergo when activated which is mediated by these autophagy and glycolytic mTOR pathways (132). AMPK is an important regulator of metabolic pathways and is able to positively regulate autophagy (133). This is through ULK phosphorylation, an important early step autophagosome protein, and also downregulation of mTOR through phosphorylation of either Raptor (134) or TSC2 (135). Regulation of AMPK can also be controlled by mTOR as it is able to inhibit ULK-AMPK interactions (133). The interaction between all these metabolic pathways provide a complex issue that needs to be addressed. Especially in the case of CD8^+^ T cell activation where metabolism regulation is important to rapid proliferation in both bacterial and allergic inflammatory contexts. Further understanding of this dynamic could open avenues for targeting T cell mediated asthma phenotypes. Activation of T cell receptors also has been demonstrated to co-activate both mTOR and autophagy pathways; however, it was also shown that autophagy was able to operate independently from mTOR (131). It has also been described that in CD8^+^ CD28^−^ T cells that TCR engagement had a decreased ability to induce autophagy, in comparison to CD28^+^ T cells, therefore making them more likely to fail their metabolic demands and senesce when activated (131). In senescent CD8^+^ human cells, p38 induced increased autophagy with no metabolic remodeling, with mTOR-independence, suggesting the possibility of a therapeutic knockdown in mediation of these rapidly proliferating cell populations in inflammatory contexts (136). Strikingly, Atg5 knockout in T cells displayed a more effector memory phenotype with more IFN and TNF production (137). In certain stages of asthma pathogenesis, the generation of memory T cells may exacerbate symptoms through an overreaction to antigen and lead to more chronic cases. Due to the interconnection of metabolic pathways and the role of autophagy in the cellular energetics of T cells, studies to identify novel therapeutic targets should be done. Targeting of autophagy through other metabolic systems, such as mTOR, may provide innovative approaches, but adverse effects caused by non-autophagy specific mechanisms could be induced.

**Table 2:** Summary table of autophagy in lymphocytes.

**Autophagy-related gene**	**Cell type**	**Function of autophagy**	**References**
Atg3	CD8^+^ T cells	Survival and mitochondrial maintenance	(104, 105)
	iNKT	Memory formation and mitochondrial maintenance	(106)
Atg5	CD8^+^T cells	Homeostasis and survival	(107, 108)
		Activation and proliferation	(107)
		Memory maintenance	(109, 110)
	CD4^+^ T cell	Homeostasis and survival	(107)
		Activation and proliferation	(107, 111)
		Memory maintenance	(112)
		FoxP3 expression	(113)
	B cells	Plasma cell survival and Ig production	(114, 115)
		Mature B cell homeostasis and survival	(116)
		Peripheral B cell homeostasis and survival	(117)
		Internalization of BCR to MHC-II vesicles	(118)
	ILC2	Homeostasis and survival	(27, 119)
		Effector function	(27)
		Metabolic homeostasis	(27)
	iNKT	Homeostasis and survival	(120)
		Effector function	(121)
Atg7	CD8^+^T cells	Homeostasis and survival	(105, 107)
		Memory maintenance	(109, 110)
	CD4^+^ T cell	Activation and proliferation	(122)
		Effector function	(122)
		FoxP3 expression	(113)
	B cells	Memory maintenance	(123, 124)
		B1a B cell homeostasis and survival	(125)
		Plasmablast differentiation	(126)
	iNKT	Homeostasis and survival	(120)

In inflammatory contexts, such as asthma, CD4^+^ T cells can play into both T_H_17 and T_H_2 type mediated inflammation (138) and have also been shown to utilize autophagy (139). Like their CD8^+^ counterparts, CD4^+^ T cells have also been found to have extensive autophagy involved in a variety of cellular functions, such as metabolism and memory (112). During activation, autophagy is massively upregulated in CD4^+^ T cells (122, 140). Multiple proteins have been described to regulate autophagy in CD4^+^ T cells, such as TNFAIP3 (141) and Vps34 (142, 143), and both play roles in their cellular metabolism. In general, most literature focuses on the increase in autophagy in CD4^+^ T cells as the need for energy is high when in activated states. Metabolic proteins and autophagy are essential to the understanding of both T cell types in inflammatory diseases; they also provide possible therapeutic targets as well. Cell specific knockout of mTOR has been demonstrated to increase autophagy and promote CD4^+^ T cell survival in highly inflamed states such sepsis (144, 145). IL-21 has also been observed to engage mTOR, therefore suppressing autophagy, in CD4^+^ T cells leading to their dysfunction in the differentiation and effector functions in systemic lupus erythematosus (146). This shows that metabolism still plays an important role in inflammatory states of infection or disease, and the massive role that autophagy plays in the maintenance of CD4^+^ T cell counts and healthy function. In the case of allergic asthma, clinical treatments targeting autophagy may be able to diminish T_H_2 polarized CD4^+^ T cell populations. Strikingly, in T_H_2 polarized CD4^+^ T cells, selective autophagy was found to prevent sustained TCR activation by targeting Bcl10 for degradation and limiting NF-κB activation (147). These results have not been tested in other CD4^+^ populations, leading to the possibility that the result is only in T_H_2 polarized cells. In regulatory CD4^+^ FoxP3^+^ T cells (Treg), autophagy is essential for maintaining healthy function (113), including suppression of these pro-allergic T_H_2 cell populations. In allergic diseases, such as asthma, it is important to realize the dual functionality of Treg populations as pro-allergic environments can skew Tregs to become more pro-inflammatory (148). Depending if Tregs from asthmatic patients are polarized to this pro-inflammatory or suppressive phenotype, targeting of autophagy in these cell populations would have to be case dependent. Targeting of autophagy in T_H_2 polarized CD4^+^ cells may be helpful if the patient has a pro-inflammatory Treg phenotype. Otherwise targeting of autophagy in CD4^+^ populations could possibly deplete suppressive Treg populations which would exacerbate inflammation and symptoms. These changes and differences between patients make determining a route of treatment difficult. In relation, there has been an association between age and overactive autophagy causing persistence of dysfunctional mitochondria in CD4^+^ T cells leading to chronic inflammation and immune system impairment (149). This suggests that even over time autophagic flux can change leading to chronic disease and inflammation as well. Due to energy metabolism varying between CD4^+^ T cells subsets and patients targeting of these cells would be complicated due to differential impact on autophagy depletion (139).

Autophagy plays a significant role in a variety of important functions of B cells and plasma cells. It is involved in B cell memory maintenance (123, 124), homeostasis, survival, and effector function (116). More specifically autophagy, Atg7, has been described to be critical in tissue resident, B1a, B cells which play a key role in antigen presentation in pulmonary disease (125). Due to plasma cell function as immunoglobulin-secreting cells, ER stress generated by this process makes them extremely reliant on autophagy for survival (114, 115). Autophagy also plays a role in B cell differentiation to plasma cells and a larger role in subsequent immunoglobulin production in autoimmune diseases, such as lupus, which could provide a possible therapeutic target the treatment of these pathologies (117, 126). Interestingly, B cell responses to different stimuli produce different cellular responses fluctuating between canonical to non-canonical autophagy (116, 150). Recently, autophagy inducers were able to restore survival of B cells in aging patients with impaired autophagy (151). Changes in autophagic flux and mechanisms make the characterization of B cell autophagy complicated. However, their involvement in a variety of different immune branches allow them to be targeted for a large impact in therapy for lung inflammatory phenotypes. The role of B cells in asthma has been demonstrated to be detrimental as they respond to T_H_2 cytokine secretion, IL-4, by upregulating autophagy, survival, IgE secretion, and enhancing antigen presentation leading to exacerbation of inflammation (152). Autophagy has also been shown to be critical for sustained inflammatory and autoimmune diseases (153) even if it is not essential for normal development of B cells (117). Secretion of IgE is primarily by plasma cells and is a major mediator in allergic response as it binds to mast cell receptors for an inflammatory response (154). Due to the critical role of autophagy in these cells, it may provide a therapeutic target. For example, specific phenotypes of disease, such as B cell activating factor from the TNF family (BAFF) induced inflammatory diseases could be targeted through regulation of intracellular Ca^2+^ level or CaMKII, AKT, or mTOR ultimately regulating autophagy and attenuating disease (155). Increased levels of BAFF have been observed in child asthma patients compared to healthy children (156). A variety of cytokines have been found to effect autophagy in B cells including IFN-α (153) and IL-4 (152). Determining which cytokines are interacting with the B cells at a patient to patient basis is essential to characterizing individual disease phenotypes. Interestingly, LAMP-2C plays a natural inhibitory role in MHC II presentation in B cells through downregulating CMA which skews presentation in response to external queues (157). Atg5 has also been established to play a role in the relocalization of internalized BCR to MHC-II containing vesicles (118). Autophagy provides a viable target for B cell mediated inflammatory disease as it plays a key role in their immunoglobulin secretion and antigen presentation. Due to their sustained inflammatory potential, they provide a great clinical avenue for treatment.

### Innate-Like Lymphocytes

**Figure 2:**
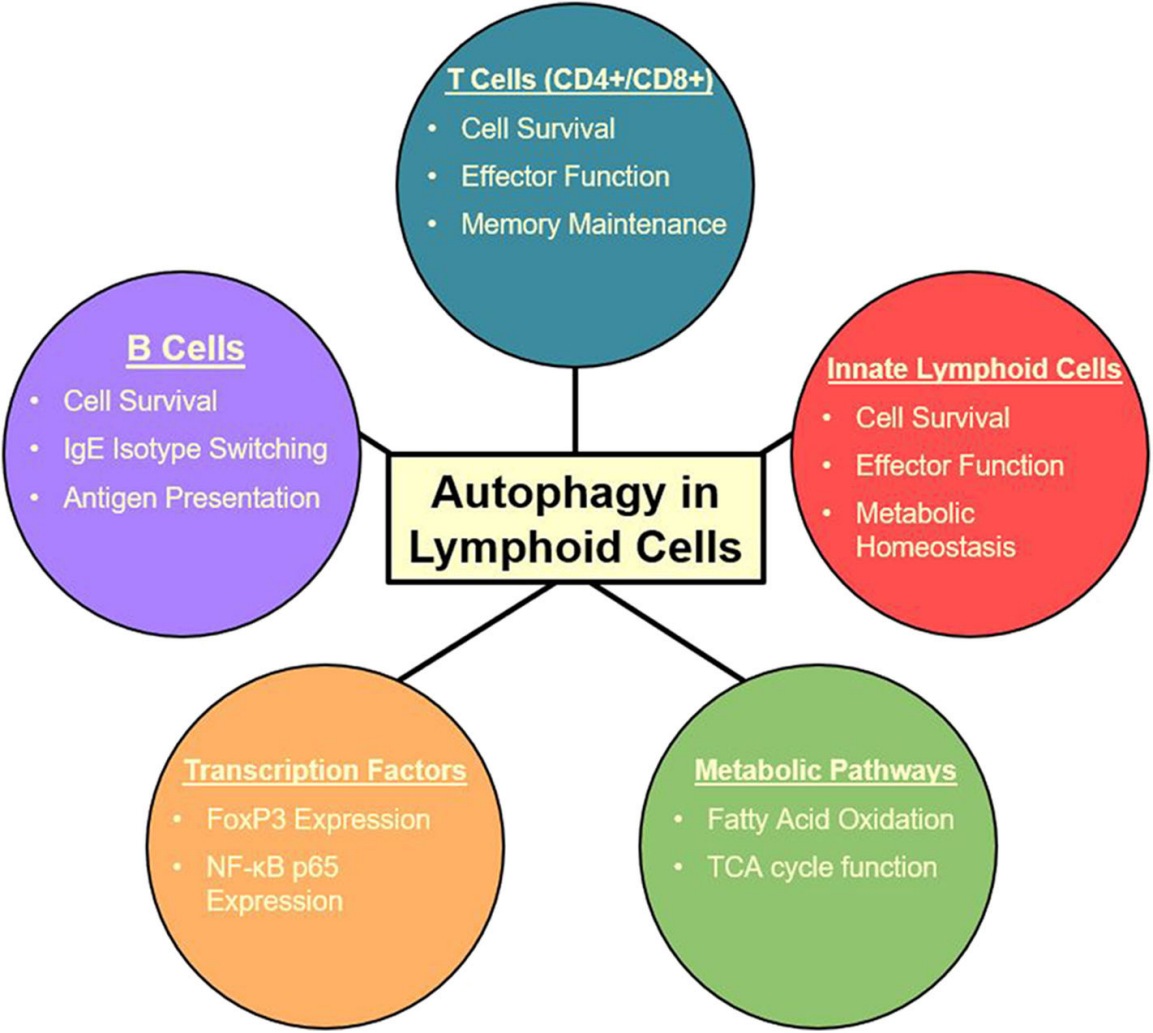
Summary of autophagy functions in the lymphoid cell lineage (27, 105, 107–115, 117, 118, 147).

Autophagy also plays a crucial role in group 2 innate lymphoid cells (ILC2) (27) which are critical players in a variety of inflammatory diseases including asthma (158, 159). ILC2s are some of the first cells to receive signals through alarmin release from pulmonary epithelial cells. With these signals, ILC2s are the first producers of T_H_2 cytokines which produce an allergic response and contribute to asthma pathogenesis (159). Specific targeting of ILC2s may be a therapeutic avenue to approach for these initial activating steps, possibly decreasing the downward cascade as well as asthma symptoms. Autophagy-deficiency, Atg5^−/−^, in ILC2s has been shown to directly affect the homeostasis and effector function of those cells. Interestingly, the lack of critical autophagic machinery impaired ILC2 ability to produce T_H_2 cytokines and lead to increases in apoptosis (27). Lower NF-κB activity also indicated less activation and cytokine secretion in Atg5 defective mice. Further analysis through Ki-67 showed that proliferation was also decreased in ILC2s in both IL-33 challenged and wild type mice. On the other hand, the induction of autophagy through the overexpression of master regulator TFEB in the Tfeb transgene (Tfeb^TG^) mice was associated with higher levels of proliferation, cytokine secretion, and activated ILC2s. Autophagy overexpression and Atg5 deletion were shown to have mirrored effects on ILC2 functions (27). Other studies have also found that Atg5 has a prevalent role in the regulation of the effector function and survival of innate lymphoid cells (119), while suppression of mTOR with inhibitors led to increase in effector function and population of innate lymphoid cells (160). These conclusions are consistent with the observations in other lymphoid cell types in which the relationship between mTOR and autophagy is dichotomous. Due to ILC2s' role as the major cytokine producers in asthma (159, 161, 162), they provide a promising target for amelioration of allergic asthma due to their tight dependence on autophagy pathways (27, 107). The multiple effects of autophagy on lymphoid cells is summarized in Figure 2. Further explorations are required to characterize the effect of autophagy inhibition during asthma pathogenesis in order to formulate a specific clinical approach.

Invariant natural killer T cells (iNKT) have also been shown to be affected by autophagic flux (120, 121) and have been described to play a key role in asthma pathogenesis (163, 164). With autophagy-deficiency, Atg5^−/−^, T_H_1 iNKTs were found to have decreased effector functions with less IL-17 and IFN-γ production when challenged with α-GalCer, an iNKT activator, *in vitro;* this finding was further supported after *in vivo* challenge with α-GalCer (121). Atg5^−/−^ has also been demonstrated to induce iNKT cell death and to disrupt cell cycle progression associated with increased mitochondrial stress in multiple iNKT subsets (120). More specifically, autophagy has been reported in later stages of iNKT development to be essential in the development to mature cells (120, 121). Other autophagy proteins, such as Atg3 has been shown to be critical for iNKT memory formation. Like other autophagy-deficient models, there was increased levels of defective mitochondria and ROS when challenged, in this case with viral infection. They further described that mitochondrial proteins BNIP3 and BNIP3L were important for memory formation and autophagy knockout led to mitochondrial disruption and loss of these essential proteins (106). Important autophagy interacting proteins like Vps34 have been found to be crucial for iNKT development past early stages, however whether this is due to its interaction between autophagy or its other interactors has yet to be investigated (165). Regardless, targeting of direct and indirect proteins in the autophagy pathway may provide a clear target for treatment to ameliorate asthma symptoms. Type 2 cytokine production by T_H_2 polarized iNKTs and other CD4^+^ T cells make them significant players in asthma pathogenesis. They have also been shown to be a significant amount of the total CD4^+^ T cell population in human asthmatic lungs, but not healthy controls (163). These iNKTs provide a very clear and present target for autophagy targeting for the amelioration of asthma.

## Role of Autophagy in Viral-Induced Lung Inflammation

Viral lung infections complicate treatments in asthma patients and can lead to treatment resistance (166). Due to the role of autophagy in clearing of intracellular components, there is a lot of interaction between autophagic and viral proteins. Often bacteria and viruses find ways to attack autophagy to avoid autophagosome formation and subsequent destruction (167); in some cases they are even able to hijack and increase autophagy at some point in infection for increased replicative potential or aid in escape (168). Ultimate suppression of autophagy occurs after chronic viral infection. Infection of APCs and subsequent suppression of autophagy in the long term can lead to activation of the inflammasome (25, 30, 79, 96), and ultimately lead to lung remodeling. Inflammatory cytokines, such as IFN-γ and IL-22 produced by innate lymphocytes stimulated by IL-1 and IL-23 (169) may condition the pulmonary airways and contribute to airway remodeling through interactions with resident cell types. Inconsistencies in autophagic flux due to viral infection in lung resident cells may contribute to asthma pathogenesis directly with these inflammatory reactions. Older studies have noted that previous viral infection predisposes patients for late asthmatic reactions (170). In support, a high percentage of patients coming into the modern clinic have previously experienced a recent respiratory viral infection (171). Viral infection of the lungs also can contribute to airway remodeling which can have lifelong effects (172, 173). Infection of epithelial and immune cells could potentially affect the autophagic flux to exacerbate airway remodeling. Production of IL-1 and IL-23 by myeloid cells with decreased autophagy (43), due to viral infection, may contribute to enhanced asthma phenotypes. It also may contribute to steroid resistant lung inflammation as observed in murine CD11c^+^ specific autophagy-deficient, Atg5^−/−^, models (30). Disruption of autophagy by upper respiratory viruses contribute to both initiation and exacerbation of asthma. However, determination of specific effects of autophagy may prove challenging as the effect on epithelial and immune cells may be different. Tissue specific ablation of autophagy due to viral infection, especially in the lung, contributes to unwanted outcomes. Prevention of viral mediated autophagy disruption is key in avoiding future asthma onset.

Autophagy modulation is present in the pathogenesis of a variety of different viruses including influenza virus and coronaviruses (CoV), each which cause significant lung inflammation (174, 175). The involvement of autophagy in the process of CoV replication seems to be controversial with multiple papers citing different results (176). These different results likely stem from differences in viruses and autophagy proteins being tested. Overall, more investigation is needed to find the specific effect of CoVs on autophagic pathways. In human CoVs infections, such as SARS-CoV, MERS-CoV, and SARS-CoV-2, the cytokine storm following infection is largely mediated by production of IL-18, IL-1, and IL-6 (177, 178). Inflammasome activation in pulmonary resident APCs leads to the production of these proinflammatory cytokines, and this may be due to the lack of autophagy induced by viral infection. MERS-CoV infection of Vero B4 cells has been shown to decrease autophagy through Belcin1-ubiquitination (179) which, in the case of in APC infection, may lead to inflammasome activation due to the impairment of autophagy pathways (25, 30, 79, 96). It is very likely that lack of autophagy, induced by viral infection, will be responsible for activation of the inflammasome in lung resident APCs and therefore, lack of autophagy would cause production of IL-18, IL-1, and IL-6 in patients with COVID-19. This observation may be supported by the fact that air pollution, particularly PM2.5, has been reported to increase mTOR, a known inhibitory pathway of autophagy, impairing M2 macrophage polarization, and is associated with high secretion of IL-6 and IL-1β (180); however, its direct involvement in decreasing macrophage autophagy has yet to be determined. Interestingly, many recent studies support the notion that mortality rate and cytokine storm in patients exposed to air pollution was significantly higher. For example, a recent study, collected air pollution data and COVID-19 mortality rate from more than 3,000 counties in the United States, found that an increase of only 1 μg/m^3^ in PM2.5 is associated with an average 8% increase in the COVID-19 death rate (181).

Production of IFN plays a crucial role in protection against viral infection, such as SARS-CoV and MERS-CoV; new studies clearly suggest that delayed induction of IFN responses from APCs contributes to the pathogenesis of disease (182). Besides epithelial cells, the only candidate that shown to be effective in IFN-α production in viral infection with SARS-CoV is pDCs (183); SARS-CoV-2 like SARS-CoV has been shown to have sensitivity to IFN (184). As pDCs are crucial in the protection against CoVs and the induction of lung inflammation leading to viral clearance, their role and function needs to be investigated in patients with COVID-19. Autophagy-deficiency, Atg5^−/−^, in pDCs has been shown to halt the production of IFN-α *in vitro* and *in vivo* (75). Therefore, assessment of autophagy among various subsets of APCs, such as pDCs, after infection needs to be investigated. Based on these observations, we anticipate that autophagy inducers, particularly at early stages of infection with SARS-CoV-2, may be able to restore functional IFN-α production by pDCs and significantly reduce viral titers (75, 184). Finally, another fact that supports the notion for role of autophagy in pathogenesis of SARS-CoV-2 is the observation that patients with worse prognosis may have pre-existing conditions in regard to autophagy. Pre-existing conditions, such as obesity, hypertension, diabetes, and coronary heart disease are often associated with lack of autophagy (185, 186); therefore, it is plausible to believe that autophagy may be responsible for higher mortality rate and outcome reported after SARS-CoV-2 infection (187).

## Role of Autophagy in Maintaining Cellular Metabolism

Due to the key role of autophagy in metabolic pathways, understanding the immunometabolism mechanisms are essential. The predominant metabolic pathways, such as AMPK, mTOR, and AKT all have downstream effects regulating autophagy and ultimately affecting the inflammatory outcome. There is evidence supporting the connection between autophagy regulation and immunometabolism in a variety of different contexts. Functions regulated by autophagy can affect multiple mechanisms, such as apoptosis, mitochondrial maintenance, switches in energy-metabolism, and proteostasis. In a variety of immune cells, dysregulation of autophagy is also associated with altered cell differentiation. In the case of macrophages, the role of autophagy due to mTOR and AMPK regulation plays a role in the metabolic balance between M1 and M2 macrophages (188). Neutrophils have also been shown to have increased glycolytic activity in autophagy-deficient, Atg7^−/−^, murine models. This enhanced glycolytic activity was coupled with disrupted differentiation; inhibition of autophagy-mediated lipid metabolism also resulted in halted neutrophil differentiation (189). B1 B cells, which are very active, have higher rates of energy consumption reliant on autophagy; Atg7 deletion leads to the selective loss of B1a B cells due to down-regulation of metabolic genes and dysfunctional mitochondria (125). This further supports the necessity for autophagy in immune cells to have maintained proteostasis and removal of bulk proteins for new protein translation for differentiation and survival (188). In inflammatory contexts, like asthma, the need for energy and efficient use of that energy is crucial for highly replicative and productive immune cell types. Dysregulation of delicate metabolism through autophagy deregulation or loss leads to loss in these cell populations. Metabolic reprogramming of ILC2s, major inflammatory cells in asthma pathogenesis, has been described to be extensively linked with autophagy. Deletion of Atg5 was shown to completely change the layout of ILC2 metabolism (27). Overall decrease in energy levels led to suppressed effector function of ILC2s as well as changes in glycolysis and fatty acid metabolism. Through metabolic and transcriptomic analysis, it was demonstrated that glycolysis was upregulated while fatty acid oxidation was inhibited in ILC2s (27). These metabolic changes led to a decrease in T_H_2 cytokine production as well as decreased asthma and AHR. Further disruption of ILC2 metabolism was found through increases in ROS and disrupted mitochondria (27). The new and immerging field of immunometabolism is going to be key in the understanding a more in depth look at disease for the future. The connections between autophagy and immune cell metabolism are extensive and will likely play a pivotal role in this understanding.

## Role of Autophagy in Asthma

Due to asthma and other inflammatory diseases being heterogeneous, targeting of autophagy must be assessed on a patient to patient basis. One of the largest problems faced in the asthmatic population is preconditioning of patients before getting treatment. This preconditioning results in a heterogeneity between patients, the most significant being remodeled lungs (40) and pulmonary viral infection (166). Both of which lead to either resistance to classical treatments or exacerbation of symptoms and further disease pathogenesis. Autophagy plays a prevalent role in a variety of airway remodeling processes (62). These processes can range from regulation of fibroblast populations and fibrosis (59) to increased levels of inflammatory cytokine secretion due to autophagy overactivation in key cell populations, such as ILC2s (27). Interactions between cells in a remodeled lung is completely different in comparison to healthy lung. There is definitely a link between deficits in autophagy due and asthma in the population (45, 46), still its ability to precondition patients before reaching clinical treatment and contribute to asthma pathogenesis has yet to be fully tested. The challenge faced is determining the level and start of lung remodeling in a patient as well as its cause. Autophagy does play a critical role in this development, however there are other factors. As such a large player in inflammatory diseases and general function of eukaryotic cells, autophagy does provide a large therapeutic target. However, determining the role it plays in different cell types is key to understanding how to specifically target it. Targeting of autophagy in the wrong cell type can lead to unwanted inflammation, such as suppression of healthy Treg populations (113) or healthy pulmonary epithelial cells (57). Specific targeting agents must be explored for targeting of certain disease phenotypes where a certain type of inflammation is present. Moreover, in order to reach the best clinical outcome, it is also crucial to consider the stage of development of the disease. Indeed, depending if asthmatic patients are in the initiation or exacerbation phases of the pathogenesis, the specific cell type to be targeted should be considered. The kinetics of disease development should then be a determining factor in identifying the therapeutic target. Interestingly, systemic targeting of autophagy may be able to overcome some of the detrimental effects it may have on a small subset of helpful cells, but the hypothesis is yet to be tested.

## Autophagy Modulators and Potential Treatments for Lung Inflammation

Autophagy dysfunction has been involved in the pathogenesis of diverse human diseases and in particular in allergic asthma and airway inflammation, and therefore, the regulation of the autophagy mechanisms has emerged as a potential approach recently. Autophagy is a process precisely regulated by a network of proteins. Among the various autophagy modulators, there are several compounds that are FDA approved. Among them, Rapamycin is an antibiotic capable of inducing of autophagy via inhibition of mTOR activity. Rapamycin binds to the cytosolic protein FKBP-12, leading the destabilization of the mTOR complex (190). This drug is used as an immunosuppressant to prevent organ transplant rejection and could represent a potent inducer of autophagy, however its clinical application as an autophagy activator requires further investigation.

Trehalose is a natural disaccharide found in organisms, such as bacteria, fungi, and plants. It has been reported to induce autophagy via mTOR-independent pathway (191). It has been previously established that trehalose can inhibit human cytomegalovirus infection in multiple cell types (192). This result suggests that autophagy inducers could also be considered as a therapeutic option against viral-induced lung inflammation in a selective manner. As increasing autophagy in the early stages of SARS-CoV-2 infection could be beneficiary, trehalose therapeutic application in this context should be explored.

Tamoxifen is a non-steroidal estrogen receptor (ER) antagonist used widely a chemotherapeutic agent against breast cancer (193). Tamoxifen treatment is known to induce autophagy (194) and many recent studies suggest that tamoxifen has antiviral function and should be use in resistant virus infections (195). Interestingly, a randomized clinical trial demonstrated that Carbamazepine, an anticonvulsant drug and autophagy inducer had high efficacy in therapy of moderate or severe bronchial asthma (196). Carbamazepine decreases inositol levels (197) and it has been shown that carbamazepine induces antimicrobial autophagy through mTOR-independent pathway (198), suggesting that autophagy induction by repurposed drug could provide an easily implementable potential therapy for some asthma phenotypes. Also, FDA-approved clonidine prescribed to treat high blood pressure, binds and activates the imidazoline receptor, leading to the decrease of the level of cAMP in cells, thus triggering autophagy (199). Recently, a robust autophagy inducer was described and reported (200); α4-viral Fas-associated death domain-like interleukin-1b-converting enzyme-inhibitory protein (α4vFLIP), is conjugated to trans-activator of transcription (TAT) for cell entry, and has been shown to significantly increase autophagy *in vitro* and *in vivo*. When compared to autophagy inducers, α4-vFLIP seems to be more specific, durable, and robust particularly when utilized *in vivo* as an antiviral agent to ameliorate viral-induced lung inflammation (201).

On the other hand, some chemical compounds can also inhibit autophagic flux, such as clomipramine, which is an FDA-approved drug used for the treatment of psychiatric disorder. Clomipramine and its active metabolite desmethylclomipramine (DCMI) induced an accumulation in autophagosomal markers and a blockage of the degradation of autophagic cargo leading to an inhibition of the autophagy process (202). DCMI is also characterized by its high cytotoxicity increasing the cytotoxic effect of conventional chemotherapeutic drugs (203).

Chloroquine, a medication primarily used to prevent and treat malaria has side effects including neurotoxicity and a change in patients' QT intervals in electrocardiograms which is associated with cardiac arrhythmias. Chloroquine is also an inhibitor viral replication and autophagic flux through inhibiting PPT1 (204) and by preventing endosomal acidification; its accumulation in the endosomes and lysosomes leads to inhibition of lysosomal enzymes that require an acidic pH and prevents fusion of autophagosomes (205). However, chloroquine treatment results in multiple cellular alterations, including the disorganization of the Golgi and endo-lysosomal networks. This large range of action of chloroquine treatments makes the exploration of its autophagy inhibitor properties difficult (205). Therefore, despite anti-viral activity, probably due to inhibition of autophagy, chloroquine treatment for COVID-19 patients reported to have little or no beneficial impact (206).

Various other compounds established regulate autophagy, and many of these molecules have demonstrated to exert beneficial effects. Even though there are many studies with encouraging results, treatments with cell type specificity are not yet available. By further characterizing the role of autophagy in specific cell populations, it could be possible to design more specific approaches aiming to modulate autophagy.

## Conclusions

**Figure 3:**
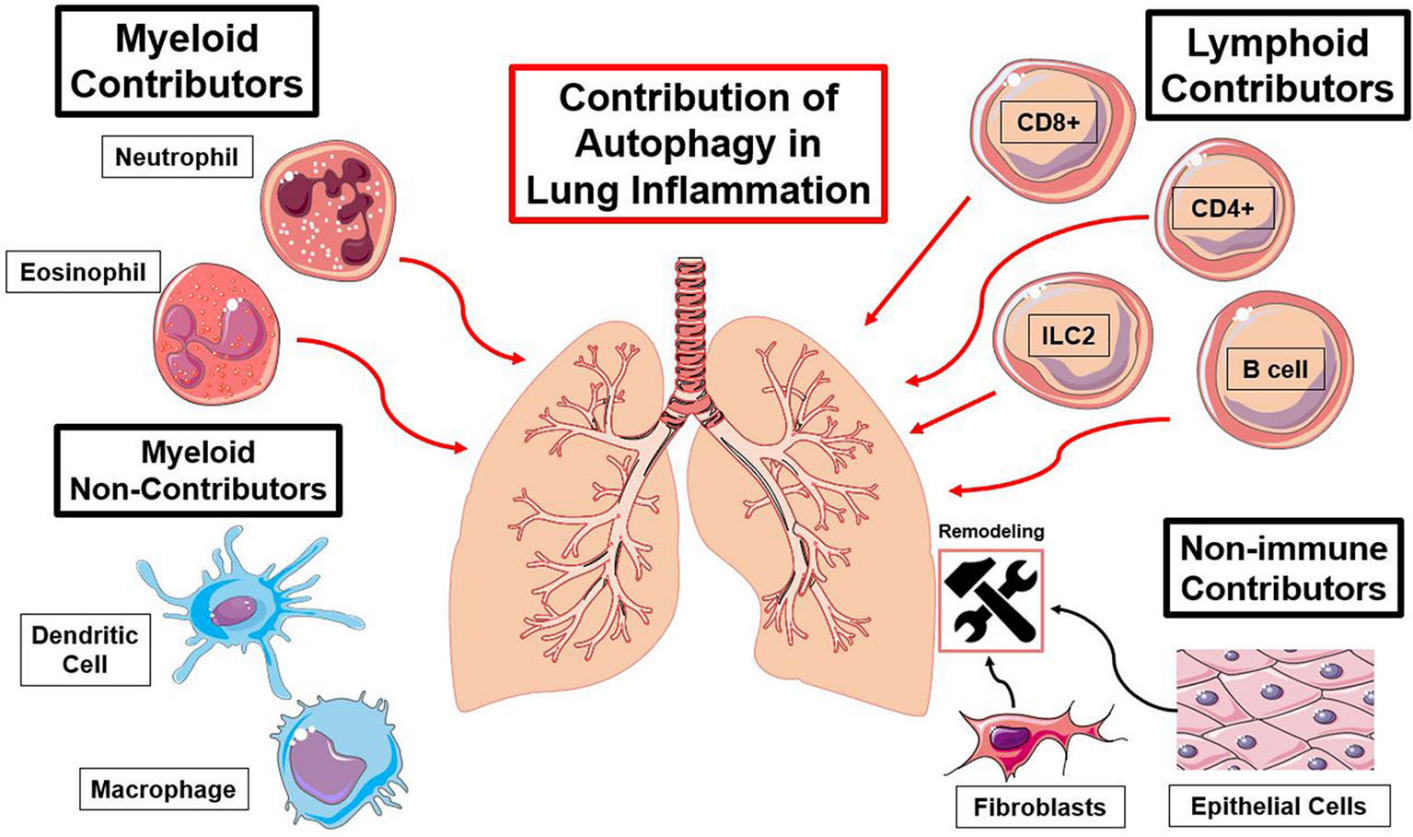
Summary of immune cell types and the functions of autophagy contributing to lung inflammation. Red lines indicate an autophagy-mediated increase in inflammation and black lines indicate an autophagy-mediated contribution to lung remodeling [(23, 25, 27, 30, 37, 41, 57–59, 63, 66, 74, 79, 89, 96, 97, 110, 127), (116, 122, 123, 137, 140, 154)].

Autophagy is a major pathway much like many other metabolic pathways, such as mTOR, and plays a massive role in cellular function. Its role in both the initiation and subsequent allergen challenge in lung inflammatory contexts needs to be explored fully. It is important clinically to understand the effect of changes in autophagic flux in specific cell types in these stages (Figure 3). Due to the heterogeneous effect of autophagy on different cell types, cell-specific targeting of autophagy must be the goal for proper clinical outcomes. Systemic targeting of autophagy provides a treatment for certain asthma phenotypes but is not ideal for all patients. Different clinical profiles would require different treatment options and a need for customized medicinal approaches. Understanding the patient's inflammatory context and environment also key in this therapeutic approach. Much like any other asthma treatment, clustering of patients based on their similarities is essential. These factors need to include stage of disease as well as the autophagy requirements. Therefore, determining these different disease phenotypes before treating a chronic patient must be done. The role of autophagy in the pathogenesis of viruses, such as SARS-CoV-2, must further be explored for insight into treatment options. The ideal customized treatment targeting autophagy modulation would consider both the cell specificity and the kinetics of the pathogenesis. The acquisition of promising cell specific agents must be investigated for their modulation autophagic flux. Systemic autophagic modulation may be helpful, however ideally control of certain problematic immune cell types may avoid unwanted side effects. Clustering of patients with lung inflammation may be able to direct treatment options. Lung inflammation and asthma are heterogeneous, so personalized, and clustered approaches may be a solution for designing appropriate autophagy modulators.

## Author Contributions

JP wrote the manuscript and prepared illustrations. LG-T assisted in edits of the manuscript and illustrations. OA supervised and edited the manuscript. All authors contributed to the article and approved the submitted version.

## Conflict of Interest

The authors declare that the research was conducted in the absence of any commercial or financial relationships that could be construed as a potential conflict of interest.
